# Induction of Noonan syndrome-specific human-induced pluripotent stem cells under serum-, feeder-, and integration-free conditions

**DOI:** 10.1007/s11626-020-00515-9

**Published:** 2020-11-02

**Authors:** Atsuko Hamada, Eri Akagi, Fumitaka Obayashi, Sachiko Yamasaki, Koichi Koizumi, Manami Ohtaka, Ken Nishimura, Mahito Nakanishi, Shigeaki Toratani, Tetsuji Okamoto

**Affiliations:** 1 Oral and Maxillofacial Surgery, Hiroshima University Hospital, Hiroshima, Japan; 2 Department of Molecular Oral Medicine and Maxillofacial Surgery, Graduate School of Biomedical and Health Sciences, Hiroshima University, 1-2-3 Kasumi, Minami-ku, Hiroshima, 734-8553 Japan; 3 TOKIWA-Bio, Inc., Tsukuba, Ibaraki Japan; 4 National Institute of Advanced Industrial Science and Technology (AIST), Tsukuba, Ibaraki Japan; 5 Laboratory of Gene Regulation, Faculty of Medicine, University of Tsukuba, Tsukuba, Ibaraki Japan

**Keywords:** Noonan syndrome, Disease-specific human-induced pluripotent stem cells, Disease modeling, Serum-free, Feeder-free, Integration-free

## Abstract

Noonan syndrome is an autosomal dominant developmental disorder. Although it is relatively common, and its phenotypical variability is well documented, its pathophysiology is not fully understood. Previously, with the aim of revealing the pathogenesis of genetic disorders, we reported the induction of cleidocranial dysplasia-specific human-induced pluripotent stem cells (hiPSCs) from patient’s dental pulp cells (DPCs) under serum-free, feeder-free, and integration-free conditions. Notably, these cells showed potential for application to genetic disorder disease models. Furthermore, using similar procedures, we reported the induction of hiPSCs derived from peripheral blood mononuclear cells (PBMCs) of healthy volunteers. These methods are beneficial, because they are carried out without invasive and painful biopsies. Using those procedures, we reprogrammed DPCs and PBMCs that were derived from a patient with Noonan syndrome (NS) to establish NS-specific hiPSCs (NS-DPC-hiPSCs and NS-PBMC-hiPSCs, respectively). The induction efficiency of NS-hiPSCs was higher than that of WT-hiPSCs. We hypothesize that this was caused by high *NANOG* expression. Here, we describe the experimental results and findings related to NS-hiPSCs. This is the first report on the establishment of NS-hiPSCs and their disease modeling.

**Supplementary Information:**

The online version of this article (10.1007/s11626-020-00515-9) contains supplementary material, which is available to authorized users.

## Introduction

Noonan syndrome (NS:MIM 163950) is an autosomal dominant inherited disorder of the RAS/MAPK signaling pathway, and it is generally characterized by peculiar facial features, short stature, congenital heart disease, mental retardation, and other characteristics (Noonan, 2006). Congenital mutations in genes such as *PTPN 11*, *SOS 1*, *RAF 1*, *KRAS*, and *BRAF* are involved in causing RAS/MAPK pathway abnormalities in NS (Roberts *et al*. 2013). That is why NS is considered a part of the group of so-called RASopathies, which are caused by mutations in genes that encode the ERK/MAPK signaling pathway. Although, in about 40% of NS patients, no mutations are observed in the RAS/MAPK pathway, RAS/MAPK pathway mutations have profound effects on cellular development. The ERK/MAPK signaling pathway is downstream of the fibroblast growth factor receptor. Disruption of the ERK/MAPK signaling pathway during embryogenesis impedes neural crest cell development and causes defects in the structure of the cardiac, craniofacial, and central nervous systems (Makishima *et al*. 2009).

Previously, we reported the induction of hiPSCs derived from peripheral blood mononuclear cells (PBMCs) (Hamada *et al*. 2020) without using serum or feeder cells (Sato *et al*. 1987; Takahashi and Yamanaka 2006; Takahashi *et al*. 2007; Yamasaki *et al*. 2013; Yamasaki *et al*. 2014) and also without virus integration (Nishimura *et al*. 2011; Nakanishi and Otsu, 2012; Yamasaki *et al*. 2016). This process eliminated the risk of activating nearby oncogenes by gene insertions and reduced the inactivation of tumor suppressor genes. The main advantage of our protocol is that disease-specific hiPSCs induced from patient-derived PBMCs have the same genetic background as the patient. To exploit this advantage, we aimed to produce NS disease-specific hiPSCs and apply them in a disease model under serum- and feeder-free conditions.

## Materials and Methods

### NS patient information

The patient in the present study was a 21-yr-old female who had been clinically diagnosed with NS in a pediatric clinic, and she was undergoing orthodontic treatment at Hiroshima University Hospital. Her phenotype included short stature, severe pulmonary stenosis (post-operation: 1 yr and 3 mo), and a facial appearance with hypertelorism and low set ears. A panoramic view showed two supernumerary teeth in each quadrate of her jaw for a total of four extra teeth.

### DNA isolation and mutation analysis with next-generation sequencing (NGS)

The patient’s genomic DNA was isolated from NS-PBMCs and NS-hiPSCs using a QIAamp® DNA mini kit (Qiagen, Valencia, CA) according to the manufacturer’s protocol. To reveal mutations, targeted resequencing was performed with MiSeq (Illumina, San Diego, CA) using a TruSight One Panel, which is designed to comprehensively cover more than 4800 genes involved in diseases according to the manufacturer’s protocol.

### Sanger sequencing

The mutations identified by MiSeq were verified by Sanger sequencing with specific primers (Table 1) designed in Primer3 (http://bioinfo.ut.ee/primer3-0.4.0/). The PCR product was purified with a PCR purification kit and sequenced directly using a CEQ8000 Beckman system (Beckman-Coulter, Brea, CA).

**Table 1.** Primers for Sanger sequence and RT-PCR

Gene name	Primer sequence
KRAS	(F) 5′-ACACAAAACAGGCTCAGGACT-3′
	(R) 5′-AACAGTCTGrATGGAGCAGG-3′
Sox2	(F) 5′-GGG AAA TGG GAG GGGTGCAAAAGAGG-3′
	(R) 5′-TIG CGT GAG TGT GGA TGG GAT TGG TG-3′
NANOG	(F) 5′-CAG CCC CGA TTC TTC CAC CAG TCC C-3′
	(R) 5′-CGG AAG ATICCC AGT CGG GTICAC C-3′
Oct3/4	(F) 5′-GACAGG GGG AGG GGA GGAGCT AGG-3′
	(R) 5′-CTT CCCTCC AAC CAG TIG CCC CAAAC-3
Rex-1	(F) 5′-CAG ATC CTAAACAGCTCG CAG AAT-3
	(R) 5′-GCG TAC GCA AAT TAA AGT CCA GA-3′
SeVdp NP	(F) 5′-AGA CCCTAA GAG GAC GAA GA-3′
	(R) 5′-ACT CCC ATG GCG TAA CTC CAT AGT G-3′
GAPDH	(F) 5′-TGA TGA CAT CAA GAA GGT GGT GAAG-31
	(R) 5′-TCC TIG GAG GCC ATG TGG GCCAT-3

### Primary culture of DPCs and infection protocol of SeVdp under serum-free conditions

Induction of DPC-hiPSCs was performed as reported previously (Yamasaki *et al*. 2016). Briefly, DPCs were cultured in a gelatin (Millipore, Billerica, MA)-coated 12-well plate at a density of 1 × 10^5^ cells in RD6F serum-free medium (Sato *et al*. 1987) and were infected with SeVdp (KOSM) (Nishimura *et al*. 2011) vector at MOI 6 once at room temperature for 2 h and then at 37°C overnight in a humid atmosphere of 95% air/5% CO_2_ in RD6F medium. Then, the infected cells were trypsinized and seeded on fibronectin (2 μg/cm^2^) (Sigma-Aldrich, St. Louis, MO)-coated 6-well plates at a density of 1.0 × 10^4^ cells in hESF9-medium (Furue *et al*. 2008; Yamasaki *et al*. 2014; Hamada *et al*. 2020) at 38°C in a humid atmosphere of 95% air/5% CO_2_. The medium was changed every other day. Both WT-DPC-hiPSCs and NS-DPC-hiPSCs were infected following the same protocol.

### Isolation of peripheral blood mononuclear cells (PBMCs) and infection protocol of SeVdp under serum-free conditions

Induction of PBMC-hiPSCs was performed as reported previously (Hamada *et al*. 2020). Briefly, PBMCs were prepared by density gradient centrifugation in a Histopaque 1077 (Sigma-Aldrich) and were cultured in RD6F serum-free medium supplemented with IL-2 (CELEUK, Takeda Pharm., Osaka, Japan) for 6 d at 37°C in a humidified atmosphere of 95% air/5% CO_2_. Then, PBMCs were infected at a density of 1 × 10^5^ cells with SeVdp (KOSM) 302 L (Nishimura *et al*. 2017), which does not integrate into the host genome, at an MOI of 6 for 2 h at 32°C in RD6F medium in a 48-well plate (BD Falcon®, Franklin Lakes, NJ) in a humid atmosphere of 95% air/5% CO_2_. The infected cells were collected by centrifugation at 200×*g* for 5 min and seeded on a Laminin-E8 (0.5 μg/cm^2^) (Nippi, Tokyo, Japan)-coated 6-well plate (BD Falcon®) in hESF9 medium at 38°C under the conditions described above. The medium was exchanged every other day. Both WT-PBMC-hiPSCs and NS-PBMC-hiPSCs were infected following the same protocol.

### Passaging of DPC-hiPSCs and PBMC-hiPSCs under serum-free conditions

Approximately 14 d after the infection of DPCs and PBMCs, we observed ESC-like colonies. These colonies were mechanically picked with a P-200 pipette (Gilson, Villiers-le-Bel, France) and further cultured in a laminin-E8-coated 4-well plate (Thermo Scientific, Waltham, MA) in hEFS9 with TGF-β1 or activin A. The hiPSCs were passaged every 5–7 d by a mechanical procedure as described above.

### Alkaline phosphate (ALP) staining

Alkaline phosphate (ALP) staining was performed using a Fast Red substrate kit (Nichirei Biosciences Inc., Tokyo, Japan) according to the manufacturer’s protocol as described previously (Yamasaki *et al*. 2014). Images of the dish were taken utilizing LUMIX (Panasonic, Osaka, Japan) and assessed for positive area using ImageJ (Abramoff *et al*. 2004). The reprogramming efficiency was determined as the number of ALP-positive colonies per total number of infected cells.

### RNA isolation and reverse transcription PCR

Total RNA was extracted with the TRIzol RNA Isolation Reagents (Thermo Scientific) according to the manufacturer’s protocol. One microgram of total RNA and high capacity RNA-to-cDNA master mix (Applied Biosystems, Carlsbad, CA) were used for cDNA synthesis. The reverse transcription PCR (RT-PCR) was performed with KOD-FX Neo (Toyobo, Osaka, Japan) employing primers described previously (Table 1). The PCR products were size-fractionated utilizing 1.5% agarose gel electrophoresis, and PCR bands were imaged on the ChemiDoc Touch Imaging System (Bio-Rad, Hercules, CA). The RT-qPCR reactions were carried out on a AiraMx real-time PCR system (Agilent, Santa Clara, CA) using FastStart Universal Probe Master (ROX) (Roche Diagnostics K.K., Tokyo, Japan). Each 10 μL reaction contained 5.0 μL of FastStart Universal Probe Master (ROX), 0.1 μL of each Universal ProbeLibrary probe (Roche) (Table 2), 0.2 μL of each primer (25 μM) (Roche) (Table 3), 0.5 μL of cDNA template (~ 25 ng/μL), and 4.0 μL of RNase-free dH_2_O. The cycle program for product amplification was as follows: 1 cycle of 95°C for 10 min (hot-start activation), followed by 40 cycles of 95°C for 30 s (denaturation), 55°C for 1 min (annealing), and 72°C for 1 min (extension).

**Table 2.** RT-qPCR primers

Gene name	Primer sequence	Universal ProbeLibrary probe
Sox2	(F) 5′-GGG GGA ATG GAC CTT GTA TAG-3′	#65
	(R) 5′-GCA AAG CTC CTA CCG TAC CA-3′	
NANOG	(F) 5′-ATG CCT CAC ACG GAG ACT GT-3′	#69
	(R) 5′-GAG GGC TGT CCT GAA TAA GC-3′	
Oct3/4	(F) 5′-CTT CGG AAG CCC TCA TTT C-3′	#60
	(R) 5′-GAG AAG GCG AAA. TCC GAA G-3′	
GAPDH	(F) 5′-AGC CAC ATC GCT CAG ACA C-3′	#60
	(R) 5′-GCC CAA TAC GAC CAA ATC C-3′

**Table 3.** List of antibodies

Antibody	Cat. no.	Antibody type	Dilution	Company
Anti-Oct3/4	MAB4401	Mouse monoclonal	1:200	Millipore
Anti-SSEA4	MC813-70	Mouse monoclonal	1:100	Stemgent
Anti-Tra-1-60	09-0010	Mouse monoclonal	1:200	Stemgent
Anti-β III tubulin	MAB3408/1/637	Mouse monoclonal	1:300	Chemicon
Anti-α-SMA	NI584	Mouse monoclonal	1:1	DAKO Cytomation
Anti-AFP	MAB1368	Mouse monoclonal	1:100	R&D
Alexa Flour® 488-conjugated goat anti-mouse IgG	A11001	Goat polyclonal	1:300	Invitrogen

### Immunocytochemistry

We studied the pluripotency of hiPSCs by immunocytochemical analyses described previously (Yamasaki *et al*. 2014). Briefly, the cells were fixed with 4% paraformaldehyde (PFA) and stained with primary antibodies against OCT4 (diluted 1/200; MAB4401, mouse monoclonal, Millipore), Tra-1-60 (diluted 1/200; 09-0010, mouse monoclonal, Stemgent®, Cambridge, MA), and SSEA-4 (diluted 1/100; MC 813-70, mouse monoclonal, R&D Systems Minneapolis, MN), and the differentiated cells were stained with antibodies against β-III tubulin (diluted 1/300; MAB3408/1637, mouse monoclonal, Chemicon), α-smooth muscle actin (N1584, mouse monoclonal, pre-diluted, DAKO Cytomation, Glostrup, Denmark), and α-fetoprotein (diluted 1/100; MAB1368, goat polyclonal, R&D Systems) (Table 3). These primary antibodies were visualized with secondary antibodies conjugated with Alexa Fluor® 488 (diluted 1/300; 11,001, mouse monoclonal, Invitrogen, Carlsbad, CA). The cell nuclei and double-stranded DNA were stained with 4′,6-diamidine-2′-phenylindole dihydrochloride (DAPI). Fluorescence images were captured using a Zeiss inverted LSM 700 confocal microscope (Carl Zeiss GmbH, Jena, Germany).

### Differentiation of NS-hiPSCs into three germ layers in vitro and in vivo

The in vitro and in vivo differentiation of WT-DPC-hiPSCs and WT-PBMC-hiPSCs was performed as described previously (Yamasaki *et al*. 2014) Hamada *et al*. 2020). To confirm the in vitro differentiation capacity of NS-hiPSCs, we performed embryoid body (EB) assay. Undifferentiated hiPSCs were cultured in hESF6 without FGF2, heparin, and TGF-b1 or activin A in low-attachment 96-well plates (Sumitomo Bakelite Co., Ltd. Tokyo, Japan) for 4–5 d, and 3–5 EBs were then transferred to gelatin-coated 35 mm dishes and further cultured for another 21 d in hESF6. The medium was changed every 3–5 d. Then, the cells were fixed and stained with the antibodies in Table 1. In vivo, NS-hiPSCs were injected into the dorsal flank of SCID (CB17/Icr-Prkdcscid/CrlCrlj) mice (1 × 10^6^ cells/100 μL of the cell suspension). Approximately 10 wk after the injection, the tumors were surgically dissected. After fixation with PBS containing 4% formaldehyde, teratomas were embedded in paraffin. Then, each section was stained with hematoxylin/eosin and Alcian blue/PAS. The histological findings were evaluated using a Nikon ECLIPSE E800 microscope (Nikon Corporation, Tokyo, Japan) and photographed with a Leica DC500 camera (Leica Microsystems AG, Wetzlar, Germany).

### Karyotyping of NS-hiPSCs

After 2 h of incubation with colcemid (Nakalai Tesque, Kyoto, Japan) at a final concentration of 0.25 μg/mL, we prepared the chromosomes using a pre-warmed (37°C) hypotonic solution (KCl 0.075 M) and fixative solution (methanol/acetic acid = 3:1). The chromosomes were spread on a glass slide and stained with Giemsa solution. Approximately 50 separate metaphase spreads of hiPSCs were examined using a Zeiss Axio Imager microscope (Carl Zeiss) and mapped.

### DNA isolation and short tandem repeat (STR) analysis

The patient’s genomic DNA was isolated from patient-derived gingival tissue and NS-hiPSCs using a QIAamp® DNA mini kit (Qiagen) according to the manufacturer’s protocol. Genomic DNA was used for PCR with Powerplex 16 system (Promega Corporation, Madison, WI) and analyzed by ABI PRISM 3100 Genetic analyzer and Gene Mapper v3.5 (Applied Biosystems).

### Statistical analysis

Statistical significance was determined utilizing Student’s *t* test. *P* < 0.05 was considered to be statistically significant.

## Results

### Missense mutation

**Figure 1.**
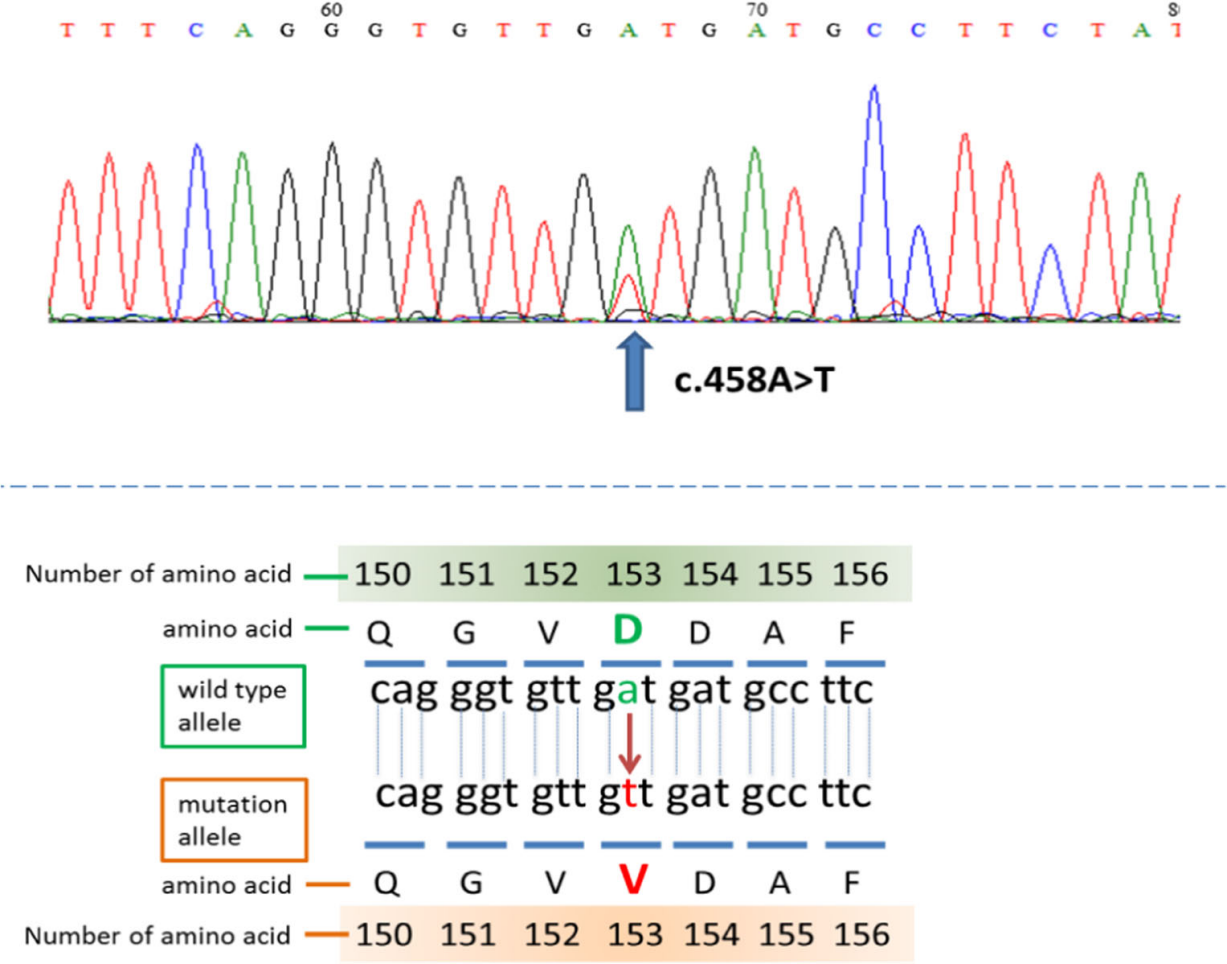
Mutation analysis. The mutation CDS 456 A > T in *KRAS* was detected with MiSeq using a TruSight One Panel and was resequenced by Sanger sequencing.

NGS analysis revealed that the patient exhibited a missense mutation in the protein coding sequence of *KRAS* (456 A > T) indicating substitution of Val for Asp at amino acid position 153 (D153V). This mutation was revealed by MiSeq using a TruSight One Panel and was further confirmed by Sanger sequencing (Fig. 1).

### High expression of *NANOG* in NS-PBMCs

**Figure 2.**
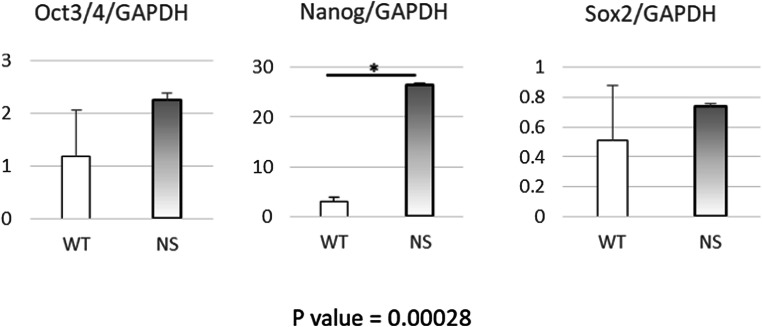
High expression of *NANOG* in NS-PBMCs. The mRNA expression of *OCT3/4*, *NANOG*, and *SOX2* in WT- and NS-PBMCs after 6-d cultivation. All data were normalized by *GAPDH* as internal control.

The NS-PBMCs cultured in RD6F serum-free medium supplemented with IL-2 showed 9-fold higher expression of *NANOG* than that of WT-PBMCs. However, no significant differences were detected in *OCT3/4* and *SOX2* (Fig. 2).

### High reprogramming efficiency and growth ability in NS-hiPSCs

**Figure 3.**
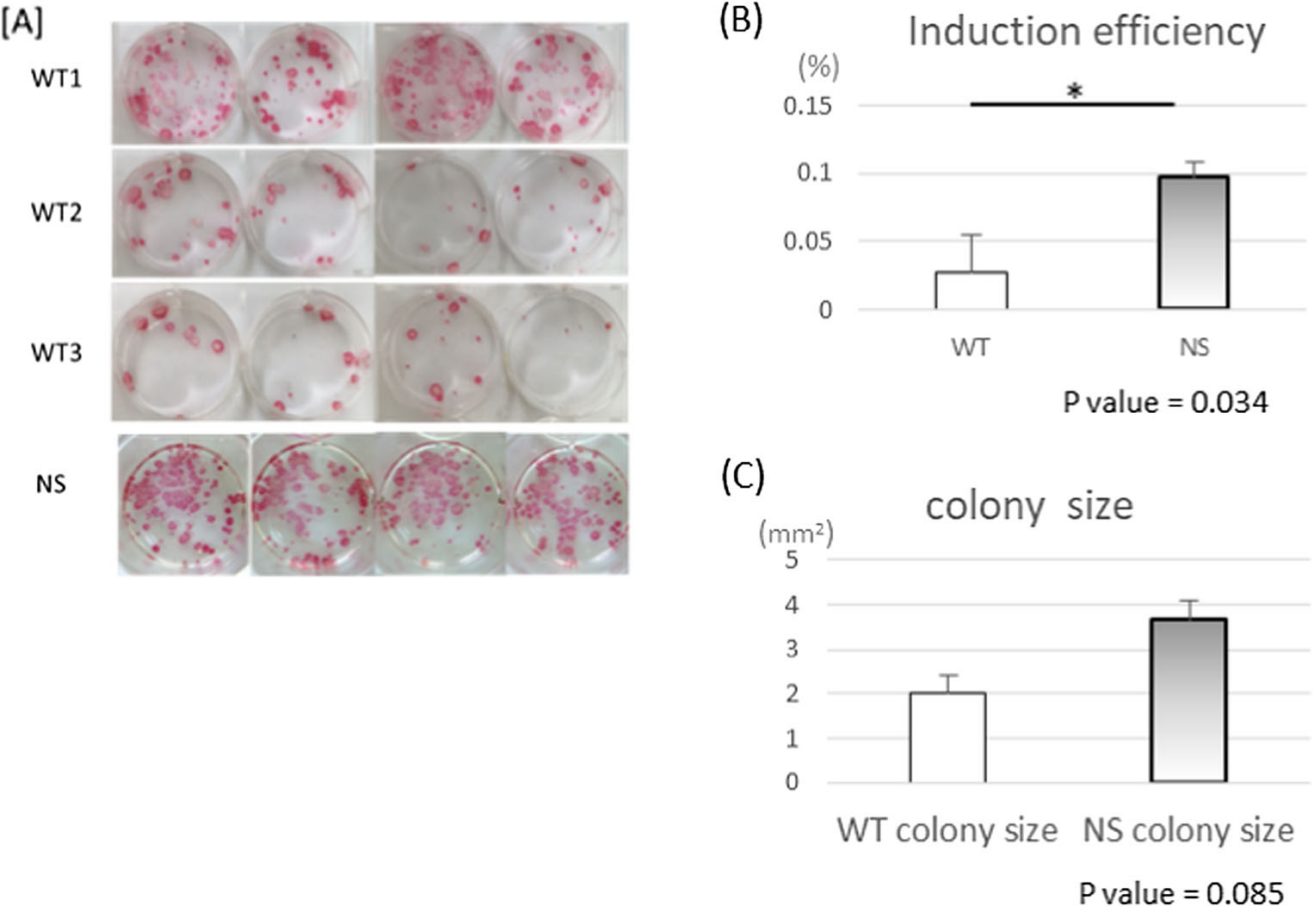
ALP-positive colonies and number of colonies. (*A*) Wells were stained with alkaline phosphate after 25-d culture. (*B*) The graph shows the induction efficiencies. The reprogramming efficiency of NS-PBMCs was significantly higher than that of WT-PBMCs. (*C*) The graph shows the colony size calculated by the following formula: ALP positive area/colony number; however, the observed difference in colony size was not significant.

The patient’s PBMCs were used for hiPSC cell reprogramming with the SeVdp (KOSM) 302 L vector at an MOI of 6 under completely feeder-free and serum-free culture conditions. The dell density of infected PBMCs was 1 × 10^5^ cells per well of a 48-well plate. After 25 d, we stained the cells in each well with an ALP staining kit (Nichirei) (Fig. 3*A*). The reprogramming efficiency was calculated as ALP-positive colonies/total number of infected cells × 100 (%). NS-PBMCs showed 3.5-fold higher reprogramming efficiency than WT-PBMCs (Fig. 3*B*). The colony sizes were not significantly different between WT-PBMC-hiPSCs and NS-PBMC-hiPSCs (Fig. 3*C*).

### Generation of integration-free NS-hiPSCs and their characterization

**Figure 4.**
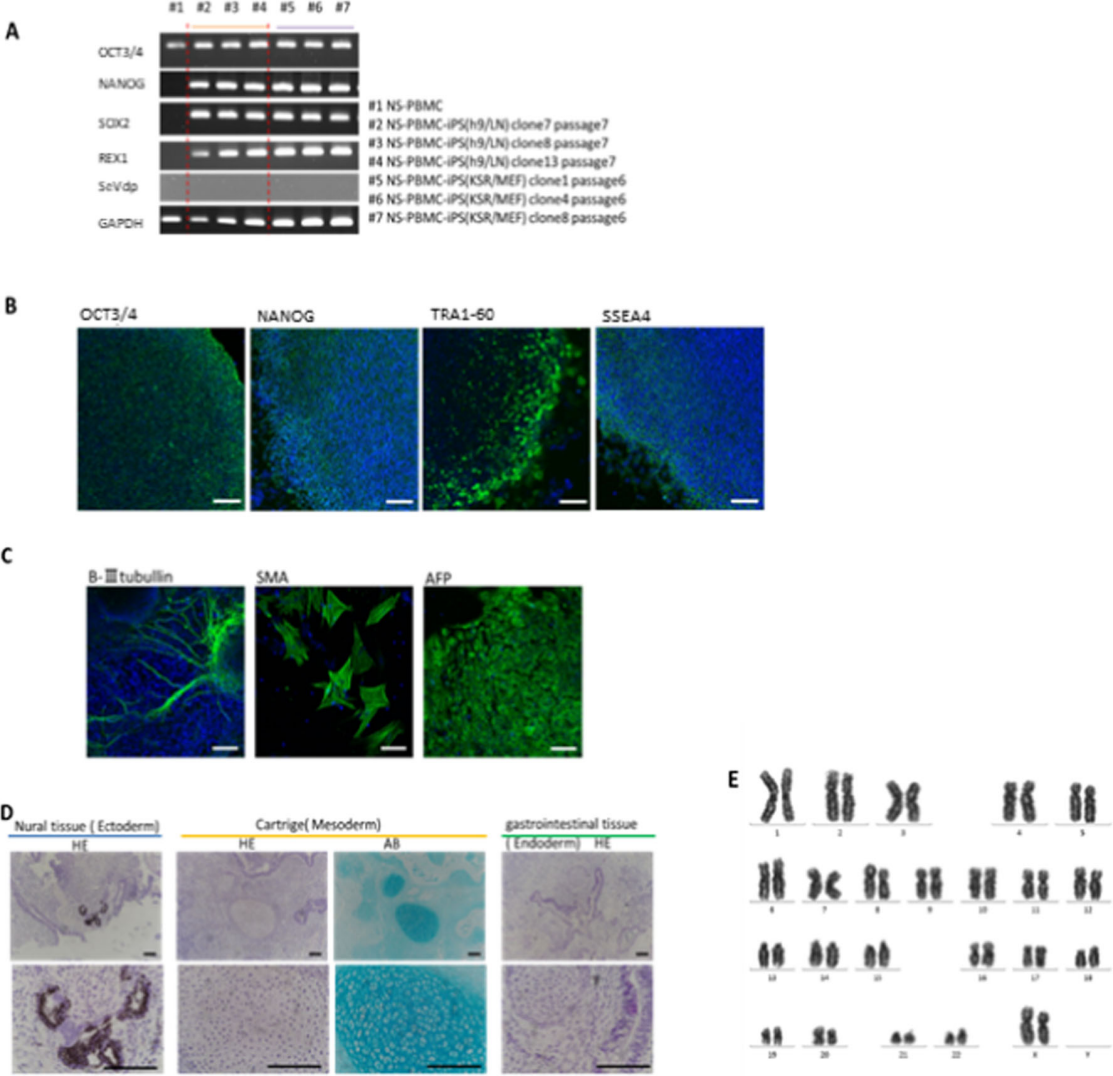
Characterization of NS-PBMC-hiPSCs. (*A*) Gene expression of pluripotent markers by RT-PCR. Although *OCT3/4* was detected before reprogramming, *NANOG*, *SOX2*, and *REX1* were expressed after reprogramming. *SeVdp* was not detected under any conditions. (*B*) Immunofluorescence staining of pluripotent markers in NS-PBMC-hiPSC clone 13 (serum-free condition) at passage 20 (OCT, NANOG, TRA1-60, and SSEA4). Each bar indicates 100 μm in length. (*C*) Immunofluorescence staining of differentiation markers in NS-PBMC-hiPSCs clone 13 at passage 19 after 3 wk of differentiation in vivo (β-III tubulin, smooth muscle actin (SMA), and alpha fetoprotein (AFP)). Each bar indicates 100 μm in length. (*D*) Established NS-PBMC-hiPSCs differentiated into three germ layers in SCID mice. Each *bar* indicates 100 μm in length. (*E*) The karyotype was normal (2*n* = 44 + XX).

We used our WT-PBMC-hiPSC induction protocol (MOI of 6, 1 × 10^5^ cells per vector, and culturing on a laminin-E8-coated 6-well dish (BD Biosciences, Falcon®) to infect NS-PBMCs. After 14–21 d of infection, we selected several colonies, checked them for mutations, and detected D153V, which was the same as that in PBMCs derived from the NS patient. These cells expressed pluripotent markers detected by RT-PCR (*OCT*, *NANOG*, *SOX2*, and *REX1*) (Fig. 4*A*) and immunofluorescence staining (OCT, NANOG, TRA1-60, and SSEA4) (Fig. 4*B*). They also differentiated into three germ layers both in vitro (Fig. 4*C*) and in vivo (Fig. 4*D*); in the teratoma assay, NS chondrocytes were hypertrophic and contained few morphological abnormalities in the cartilage matrix (Fig. 4*D*). NS-hiPSCs at passage 20 exhibited a normal karyotype of 46, XX (Fig. 4*E*). STR results showed that NS-hiPSCs were identical to NS patient gingiva (Supplementary data).

## Discussion

Although there was no significant difference in colony size between WT-PBMC-hiPSCs and NS-PBMC-hiPSCs, the induction efficiency of NS-PBMC-hiPSCs was higher than that of WT-PBMC-hiPSCs. Further, *NANOG* expression in NS-PBMCs was higher than that in WT-PBMCs. *NANOG* plays a central role in maintaining pluripotency and cooperating with *OCT4*, *SOX2*, and other pluripotency factors. Hayashi *et al*. reported that the human *NANOG* homeodomain (hNANOG HD) was bound to *OCT4* promoter DNA, which revealed amino acid residues involved in DNA recognition using the crystal structure (Hayashi *et al*. 2015). There is a possibility that higher expression of *NANOG* in NS-PBMCs results in higher induction efficiency of NS-PBMC-hiPSCs. However, further studies are needed.

In the teratoma assay NS chondrocytes were hypertrophic and contained few morphological abnormalities in the cartilage matrix. This may be related to a well-known paradox that Ras signal enhancement promotes cell proliferation but inhibits proliferation and maturation in cartilaginous tissues (Horton *et al*. 2007). To prove this, we are trying to establish a chondrocyte induction protocol under completely serum-free conditions based on our system, which is more suitable to elucidate the cellular responses and clarify the molecular mechanism of disease under defined conditions to further understand NS pathogenesis. Moreover, Fukuta *et al*. recently developed a protocol to induce neural crest cells from human pluripotent stem cells (Fukuta *et al*. 2014). Combination with that protocol would be a powerful tool to elucidate the pathophysiology of NS and related diseases at the molecular and cellular levels, which will lead to the development of new therapeutic procedures.

## Conclusions

Here, we describe the successful generation and differentiation of NS disease-specific hiPSCs under serum- and feeder-free conditions. These NS disease-specific hiPSCs will be a powerful tool to elucidate the mechanism of disease occurrence and to develop treatments.

## Supplementary Information

ESM 1 (DOCX 165 kb)
