# Supplementary figures and images for: Induction of Noonan syndrome-specific human-induced pluripotent stem cells under serum-, feeder-, and integration-free conditions
Source: In Vitro Cell Dev Biol Anim. 2020 Nov 2;56(10):888–95. doi: 10.1007/s11626-020-00515-9 (PMC7723931; doi:10.1007/s11626-020-00515-9)

Supplementary Table. Hamada A. et al.


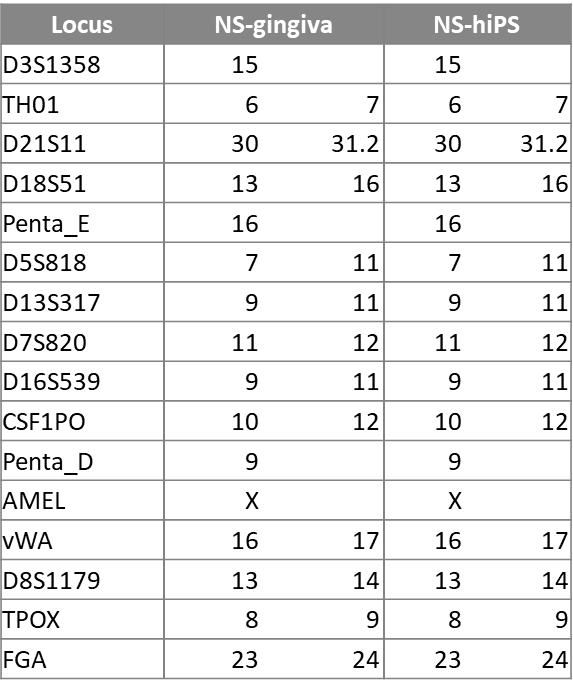

Supplement: Supplementary file 1 — (DOCX 165 kb) [file 11626_2020_515_MOESM1_ESM.docx]
